# Impact of GTV-CTV margin and other predictors on radiation-induced dysphagia in head and neck cancer patients from DAHANCA group

**DOI:** 10.2340/1651-226X.2025.44021

**Published:** 2025-09-18

**Authors:** Sarah Wordenskjold Stougaard, Ruta Zukauskaite, Richard Röttger, Ebbe Laugaard Lorenzen, Maximilian Lukas Konrad, Simon Long Krogh, Camilla Panduro Nielsen, Jeanette Frieda Aviaya Sommer, Jørgen Johansen, Jesper Grau Eriksen, Camilla Kjaer Lonkvist, Jeppe Friborg, Carsten Brink, Christian Rønn Hansen

**Affiliations:** a Laboratory of Radiation Physics, Odense University Hospital, Odense, Denmark; b Department of Clinical Research, University of Southern Denmark, Odense, Denmark; c Department of Oncology, Odense University Hospital, Odense, Denmark; d Department of Mathematics and Computer Science, University of Southern Denmark, Odense, Denmark; e Danish Centre for Particle Therapy, Aarhus University Hospital, Aarhus, Denmark; f Department of Oncology, Aarhus University Hospital, Aarhus, Denmark; g Department of Experimental Clinical Oncology, Aarhus University Hospital, Aarhus, Denmark; h Department of Oncology, Copenhagen University Hospital Herlev, Herlev, Denmark; i Department of Clinical Oncology, Rigshospitalet, Copenhagen, Denmark

**Keywords:** Radiotherapy, side effects, head and neck cancer, guidelines, modelling

## Abstract

**Background and purpose:**

This multicentre, retrospective study aimed to develop a predictive model for radiation-induced dysphagia in head and neck cancer patients, focusing on the role of gross tumour volume (GTV) to high dose CTV (CTV1) margin size and dose-related factors. Unlike previous studies focused on peak or single time-point dysphagia, this study modelled symptom trajectories using repeated follow-up data for a more complete picture.

**Patient/material and methods:**

Between 2010 and 2015, 1,948 patients with pharyngeal or laryngeal squamous cell carcinoma received definitive intensity-modulated radiotherapy (IMRT) at three Danish centres. Data included physician-rated dysphagia (grade 0–4), tumour and treatment characteristics, and AI-based segmentations of organs at risk (OARs). Predictors included GTV-CTV1 margin size, mean doses to the oral cavity and pharyngeal constrictor muscles (PCM), GTV volume, chemotherapy, tumour site, fractionation, nimorazole, sex, smoking status, baseline dysphagia, and age. A logistic ordinal mixed-effects model was fitted with patient ID as random effect. Data were split into training (70%) and test (30%) sets. Model performance was assessed using calibration plots and area under the curve (AUC).

**Results:**

After excluding incomplete cases, 1,685 patients (7,829 visits) were analysed. GTV-CTV1 margin size was not significantly associated with dysphagia, although larger margins correlated with higher OAR doses. Higher doses to the lower PCM (odds ratio [OR] = 1.30 per 5 Gy) and oral cavity (OR = 1.32 per 5 Gy) increased risk. The model demonstrated good calibration and robust discrimination (AUC = 0.77–0.84).

**Interpretation:**

Radiation dose to the oral cavity and lower PCM were the strongest modifiable predictors of dysphagia risk. Margin size was not independently associated, possibly due to confounding by clinical judgement.

## Introduction

In Denmark, approximately 1,400 patients are diagnosed with head and neck (HN) cancer each year, with an estimated prevalence of 17,000 (the Danish Head and Neck Cancer Group (DAHANCA): www.dahanca.dk). Due to the anatomical complexity of the HN region, radiotherapy (RT) is a suitable option for organ-preserving treatment. Although RT is highly effective, it is also associated with a substantial risk of side effects, which significantly impact quality of life and long-term morbidity [1, 2].

The risk of radiation-induced toxicity largely depends on the radiation dose delivered to the surrounding organs at risk (OARs) [3]. Consequently, minimising dose exposure to OARs without compromising tumour control has become a key focus in modern RT planning. Technological advancements have increased the flexibility in dose distribution planning and delivery, allowing for selective sparing of specific OARs [4, 5]. However, complete avoidance of OAR exposure remains challenging and sparing certain structures may come at the expense of others.

Besides doses to the OARs, numerous factors have been proposed to influence the risk of developing radiation-induced dysphagia, including tumour volume, chemotherapy, sex, smoking status, tumour site, radiosensitiser use, patient age, fractionation schedule, and the applied margin from gross tumour volume to clinical target volume (GTV-CTV) [6–8]. Among these, the GTV-CTV margin is of particular interest, as this is a modifiable factor, and reducing the margin has been reported to significantly lower the risk of both acute and late toxicities, including dysphagia, without compromising loco-regional control or survival [7, 8]. However, these findings from Al-Mamgani et al. were derived from a single centre cohort treated using fixed margin sizes at two different periods. Consequently, the relevance of margin size as a risk factor for dysphagia remains unclear in broader, heterogeneous, real-world patient populations where margins may vary across institutions, clinicians, and depending on tumour location, stage, or patient anatomy. Moreover, most prior studies have focused on single time points (e.g. 6 or 12 months post-treatment) or peak dysphagia scores within a defined follow-up period, which may not capture the full trajectory of dysphagia development and recovery over time [9]. This limitation is further highlighted by studies where strict inclusion criteria significantly reduce the evaluable patient population, such as a recent review of videofluoroscopic swallow studies where 45% of the identified studies were excluded due to unavailable data, comorbidities, recurrence, or other confounding factors, limiting the generalisability to real-world populations [10]. Thus, there is a need for further investigation into the real-world implications of margin variability on dysphagia risk throughout the entire post-treatment period. Until 2013, variation in the applied GTV–CTV margin for head and neck (HN) cancer (0–10 mm) was practiced across Danish centres in accordance with DAHANCA RT guidelines [11]. These variations were primarily based on different preferences across departments, with some centres typically using 0 mm and others using 10 mm GTV-CTV margin. In 2013, DAHANCA introduced a national guideline standardising the margin to a 5 mm GTV to high dose CTV1 [12, 13]. This natural variation prior to standardisation presents a unique opportunity to evaluate the impact of GTV-CTV margin size on post-treatment toxicity in a real world, multicentre cohort, where patients are otherwise comparable and treated with curative intent under the same protocol.

While the primary impact of the GTV–CTV margin on the risk of dysphagia is presumed to be mediated through increased radiation doses to OARs, this may not fully capture the complexity of treatment-related swallowing dysfunction [11]. Swallowing is a highly intricate physiological process involving a coordinated interplay of multiple muscle groups and neural pathways. Consequently, radiation exposure to tissues beyond the conventionally defined OARs, such as muscles and connective structures surrounding the high-dose target volumes, may contribute to dysphagia in ways not accounted for by standard OAR doses. The current study, therefore, explores whether the GTV–CTV margin in itself plays an additional role in dysphagia risk, independent of, or synergistic with, OAR dose, potentially by irradiating these functionally important but less well-characterised peripheral pharyngeal tissues.

The aim of this study is to develop a predictive model to gain better understanding of the contributors to radiation-induced dysphagia in HN cancer patients, focusing specifically on the added role of GTV-CTV1 margin size in addition to the dose-related factors. Unlike previous studies, which have typically assessed peak dysphagia or focused on single post-treatment time points, this study models dysphagia trajectories over time using repeated follow-up data, providing a more comprehensive picture of symptom development and recovery.

## Patients/material and methods

This multicentre, retrospective, longitudinal cohort study was based on real-world clinical data from three Danish centres. The study adheres to the STROBE (Strengthening the Reporting of Observational Studies in Epidemiology) guidelines for observational cohort studies (see Supplementary Table 1) [14].

### Patients and treatments

All patients treated with curatively intended RT at three DAHANCA centres between January 2010 and December 2015 were included, resulting in a total cohort of 1,948 patients with pharyngeal or laryngeal HN squamous cell carcinoma (HNSCC). The cohort included patients who received primary treatment with intensity-modulated RT (IMRT) and completed an RT regimen delivering at least 66 Gy. Patients were followed up for up to 5 years after the end of treatment, according to the DAHANCA follow-up routine. Patients with synchronous HNSCC were excluded. Additionally, patients with a known diagnosis of SCC of the lung or oesophagus within 3 years before or after RT for human papillomavirus (HPV) p16-negative HNSCC were excluded. Further details on treatment planning have been published by Zukauskaite et al. [13].

### Data

Data on dysphagia and patient- and tumour characteristics were available from the DAHANCA database [15]. Furthermore, information on doses to the OARs was collected from the RT treatment plans using the national DICOM database, DcmCollab [16, 17]. Dysphagia was physician-graded using DAHANCA’s categorical, ordinal scale from 0 to 4: (0) no dysphagia; (1) symptomatic, able to eat regular diet; (2) symptomatic and altered eating/swallowing, soft food; (3) symptomatic and altered eating/swallowing, only fluid food; (4) severely altered eating/swallowing; tube feeding or hospitalisation; urgent intervention indicated. As baseline dysphagia was not systematically recorded, dysphagia grade at the first treatment week was used as a proxy. If missing, the value from the second treatment week was used. Dysphagia was graded on the same 0–4 scale as described above.

An artificial intelligence (AI)-based segmentation method, previously validated, was employed to delineate OAR consistently [18, 19]. The segmentations were validated in three steps: initially, OAR segmentations of 150 patients’ CT-scans were processed by the AI model to identify potential technical issues. Then, HN experts manually validated 15 scans to ensure accuracy before applying the model to the entire cohort. Lastly, an in-house developed outlier detection tool based on principal component analysis (PCA) was used on a subset of the data to ensure quality [20].

The primary variable of interest for dysphagia was GTV-CTV1 margin size. At the same time, secondary predictors included baseline dysphagia, mean doses to oral cavity and pharyngeal constrictor muscles (PCM), GTV volume, chemotherapy, tumour site, fractionation, radiosensitiser (nimorazole), sex, smoking status, and age. GTV volume was log-transformed to reduce skewness. Nimorazole treatment was defined as having been initiated, regardless of whether the full planned dose was completed.

**Figure 1:**
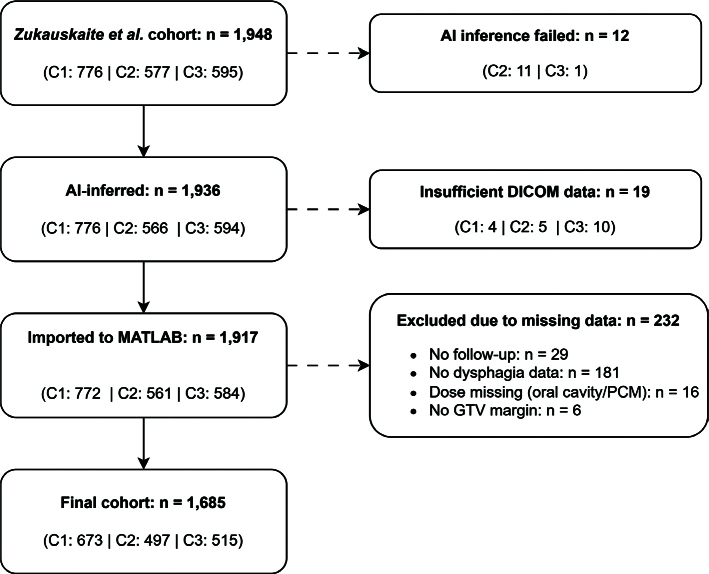
Patient selection flowchart for the final study cohort. Flow diagram of patient inclusion and exclusion. The initial cohort comprised 1,948 patients from the study by Zukauskaite et al. dataset [13]. Subgroup counts are indicated by centre (C1–C3).

Patients for whom the AI failed to generate OAR segmentations or who had incomplete DICOM data were excluded, as were those lacking any dysphagia assessment or follow-up visits. Patients without a recorded GTVCTV1 margin or without dose information for the oral cavity or PCMs were also excluded . See Figure 1 for further details.

### Statistical analyses

Patient characteristics were summarised as counts and percentages for categorical variables and means with standard deviation for continuous variables.

Ordinal logistic regression using mixed effects was used to analyse dysphagia severity over time, accounting for patient-specific differences and variability in the number of measurements [21–23]. Patients who contributed at least one dysphagia assessment during follow-up were retained. If a patient ceased follow-up before 5 years, their available visits were included up to the last completed visit and treated as missing thereafter. Dysphagia assessments were treated as repeated measures, but time was not explicitly modelled as a variable. Rather, the model estimated overall associations between variables and dysphagia severity across all available follow-up visits. This allowed to incorporate all available data points per patients while accounting for within-patient correlation.

Dysphagia grades were treated as ordinal categories, and the model estimated the probability of being at or above each grade. Patient ID was included as a random effect to account for repeated measurements and variable numbers of follow-up visits, while all clinical and dosimetric covariates were included as fixed effects. The dataset was split into a training set (70%) and a test set (30%) using centre-stratified random sampling to evaluate predictive performance.

**Figure 2:**
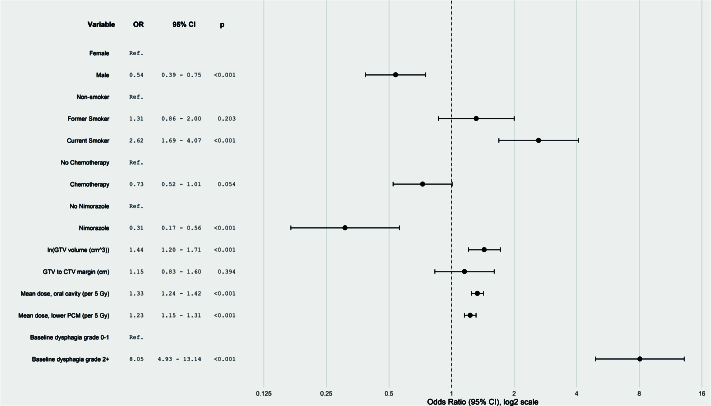
Forest plot. Forest plot of variables associated with the risk of dysphagia, displaying OR with 95% CI and corresponding p-values. The vertical dashed line at OR = 1 denotes no effect. An OR greater than 1 indicates an increased risk of higher-grade dysphagia, whereas an OR less than 1 indicates a decreased risk. The reference category for each variable is noted as ‘Ref’.

To select which covariates to include in the model, forward stepwise selection was performed using likelihood ratio tests with a significance threshold of *p* < 0.1 to identify the most parsimonious model. The GTV-CTV1 margin size was retained in all models, regardless of statistical significance, as it is the primary parameter for this study. Predictions incorporated simulated random intercepts sampled from a normal distribution with a mean of zero and a variance equal to the estimated random effect variance from the training model. Significant predictors in the final ordinal logistic mixed effect model with variables chosen by the forward selections processes were visualised in a forest plot (Figure 2) showing odds ratios (ORs) and 95% confidence intervals (CIs).

For patients with missing dysphagia assessments at both the first and second treatment visits, baseline dysphagia was imputed as 0, assuming no symptoms in the absence of recorded evidence. For modelling purposes, baseline dysphagia was dichotomised as grade 0–1 versus grade 2+ to account for highly unbalanced group sizes.

Ordinal logistic regression models with random effects were fitted using cumulative link mixed models, which account for the ordered nature of the dysphagia outcome and the repeated measurements per patient. These models were implemented in R version 4.4.1 using the clmm function.

Analyses were also stratified by time since treatment (≤ 1 year, 1–2 years, > 2 years) to explore whether associations differed across follow-up intervals.

Model performance was evaluated using area under the curve (AUC) to assess discriminative ability and using calibration plots. For these purposes, dysphagia was dichotomised into grade 2+ and grade 3+. The calibration plots were performed by grouping patients into deciles based on predicted risk and observed proportions were compared to predicted probabilities (including sampled random effects).

Finally, sub-analyses were conducted to explore the relationship between GTV-CTV1 margin sizes and mean doses to OARs based on the hypothesis that larger margins would result in higher OAR doses. For this purpose, the margin size was categorised as < 2.5, 2.5–7.5, and ≥7.5 mm. Group differences in dose were tested using the Wilcoxon rank-sum test.

## Results

Of the initial cohort of 1,948 patients, a total of 1,685 patients with sufficient information for analysis were included (See Figure 1 for further details). The cases with the highest outlier scores flagged by the PCA-based quality assurance tool were reviewed. While some deviations were observed, the overall delineations were visually acceptable. None appeared large enough to substantially impact dose estimation. As the analyses rely on mean dose to the OARs, which is more robust to such contouring variations than, for example, minimum or maximum dose, all segmentations were retained for analysis.

Patient characteristics are summarised in Table 1. A GTV-CTV1 margin < 2.5 mm was observed in 16% of patients, 2.5–7.5 mm in 54% of patients, and > 7.5 mm in 30% of patients; the median margin size was 6.1 mm (IQR: 4.4–9.3 mm). See Supplementary figure 1 for distribution.

**Table 1:** Patients’ characteristics (*N* = 1,685).

Characteristic	*N* (%) / Mean (SD)
Sex	
Female	419 (25%)
Male	1,266 (75%)
Smoking	
Never	249 (15%)
Former	741 (44%)
Current	626 (37%)
*Missing*	*69 (4%)*
Centre	
1	673 (40%)
2	497 (40%)
3	515 (31 %)
Chemotherapy	
No	834 (49%)
Yes	851 (51%)
Nimorazole	
No	129 (8%)
Yes	1,556 (92%)
Site	
Pharynx	1,296 (77%)
Larynx	389 (23%)
Fractions	
66–68 Gy	1,497 (89%)
76–78 Gy	155 (9%)
*Missing*	*33 (2%)*
GTV-CTV-margin	
< 2.5 mm	272 (16%)
2.5–7.5 mm	910 (54%)
> 7.5 mm	503 (30%)
GTV volume, median (interquartile range)	18.1 [8.1–33.7]
Mean dose, mean (SD)	
Pharyngeal constrictor muscle upper	51.9 Gy [13.8]
Pharyngeal constrictor muscle mid	58.5 Gy [8.6]
Pharyngeal constrictor muscle lower	52.8 Gy [12.7]
Oral cavity	39.0 Gy [14.4]
Baseline dysphagia	
Grade 0	1,335 (79%)
Grade 1	202 (12%)
Grade 2	110 (7%)
Grade 3	25 (1%)
Grade 4	13 (1%)
Performance status	
0	853 (51%)
1	416 (25%)
2	134 (8%)
3	10 (1%)
Missing	272 (16%)
Stage	
1	84 (5%)
2	335 (20%)
3	1 (0%)
4	0 (0%)
5	308 (18%)
6	857 (51%)
7	79 (5%)
8	1 (0%)
9	20 (1%)

SD: standard deviation; GTV: gross tumour volume; CTV: clinical target volume.

Mean doses to the middle and lower PCMs showed a pattern of higher doses with increasing margin size. In contrast, oral cavity dose was significantly higher in the small margin group (< 2.5 mm: 41.1 Gy) compared to both the 2.5–7.5 mm (39.6 Gy) and > 7.5 mm (36.7 Gy) groups. For the upper PCM, mean dose was significantly higher in the 2.5–7.5 mm group (52.9 Gy) than in the > 7.5 mm group (48.9 Gy).

The median follow-up time was 4.0 years (IQR: 2.2–4.4 years) and patients had a median of 6 follow-up visits (IQR: 4–9 visits). As shown in Table 2, higher dysphagia grades were recorded over fewer follow-up visits per patient on average.

**Table 2:** Dysphagia distribution across follow-up visits.

Dysphagia grade	Patients with grade at any visit	% of patients (*N* = 1,685)	Total visit with grade	% of all visits (*N* = 10,955)
**0**	1,235	73.3%	5,160	47.1%
**1**	1,163	69.1%	3,298	30.1%
**2**	458	27.2%	971	8.9%
**3**	149	8.8%	283	2.6%
**4**	93	5.5%	169	1.5%

Distribution (number and percentage) of patients who experienced each dysphagia grade at any follow-up visit, as well as the percentage of total follow-up visits where each grade was recorded. A total of 10,955 follow-up visits were recorded for 1,685 patients. Median number of follow-up visits per patient was 6 (IQR: 4–9). Patients may be represented in more than one category.

Forward stepwise selection resulted in the following variables to be included in the final ordinal logistic mixed effects model as fixed effects: baseline dysphagia, smoking status, (log-transformed) GTV volume, mean doses to the oral cavity and lower PCM, sex, nimorazole, chemotherapy, and GTV-CTV margin. Patient ID was included as a random effect.

**Figure 3:**
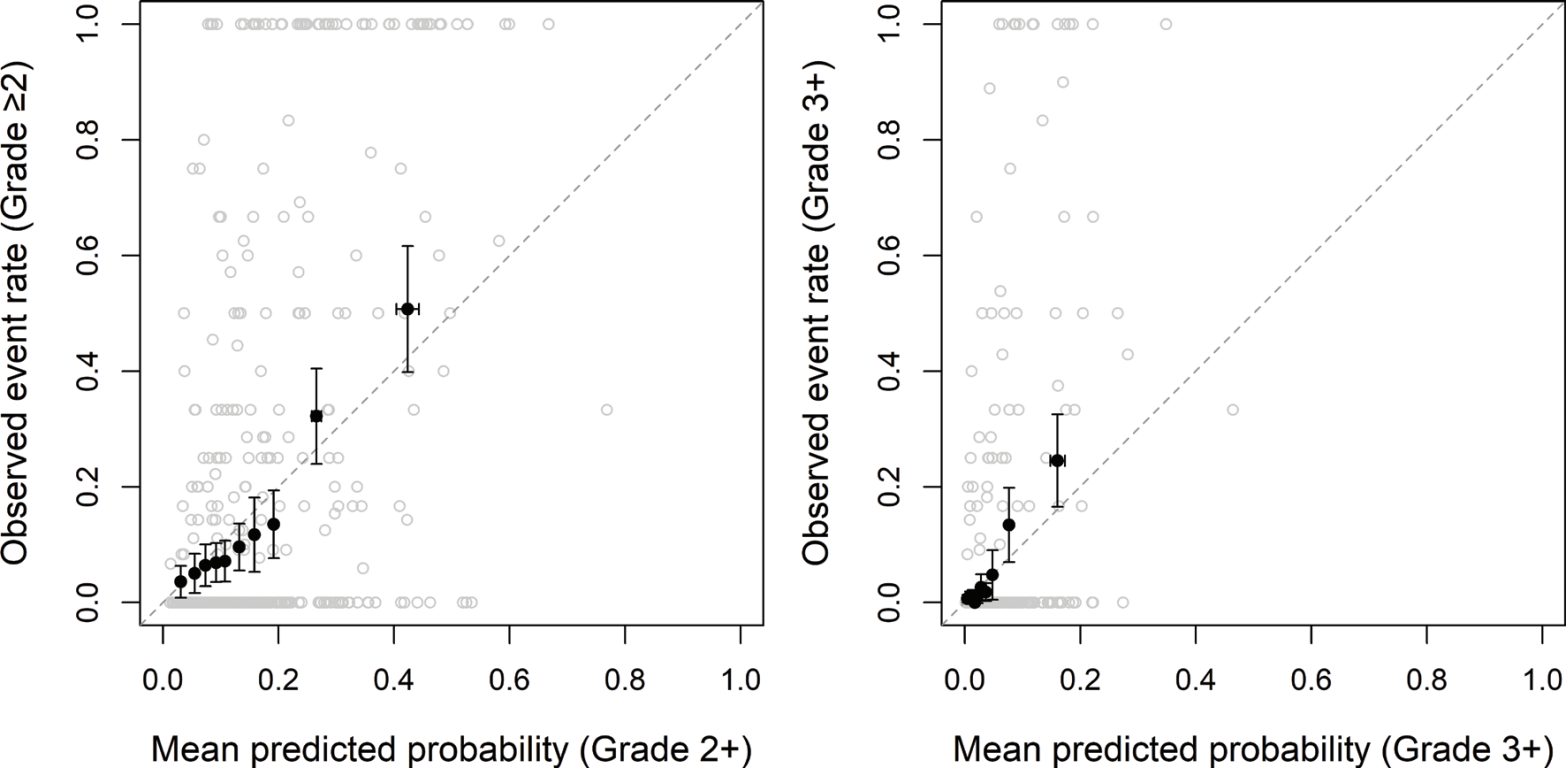
Calibration plot: Dysphagia grade 2+ and 3+ on test data. The patients were grouped into 10 equally sized groups (filled black circles), and 95% CIs are displayed in the error bars. Open grey circles represent raw data. Because dysphagia was assessed repeatedly over time, each patient’s outcome is expressed as the proportion of follow-up visits with grade 2+ dysphagia. For example, a patient with dysphagia at 1 out of 4 visits would appear at 0.25 on the y-axis.

Calibration plots (Figure 3) for predicting dysphagia at grade 2+ and 3+ showed that the observed event rates closely followed the diagonal reference line, suggesting good agreement between predicted and observed probabilities. Calibration plots for the training data (Supplementary Figure 2) showed similar patterns, supporting that the model was correctly specified.

The AUC for predicting grade 2+ dysphagia was 0.77 (95% CI: 0.75–0.78) in the training set, and 0.77 (0.74–0.79) in the test set. For grade 3+ dysphagia, AUCs were 0.79 (0.76–0.82) and 0.84 (0.81–0.88), respectively, indicating stable discriminative performance between datasets.

As visualised in the forest plot, significant predictors for increased risk of higher-grade dysphagia included baseline dysphagia grade 2+, current smoking status, (log-transformed) GTV volume, and higher mean doses to the oral cavity and lower PCM (Figure 2). Conversely, male sexand nimorazole were associated with a significantly lower risk of higher-grade dysphagia. Chemotherapy showed a trend towards reduced risk (OR = 0.71, 95% CI: 0.62–1.01), although this did not reach statistical significance (*p* = 0.54). The GTV-CTV1 margin was not significantly associated with dysphagia risk (OR: 1.15, 95% CI: 0.83–1.60, *p* = 0.39), suggesting no additional effect of the GTV-CTV1 margin on top of the dose to the OARs.

When stratifying by time since treatment, the overall pattern of associations remained consistent across post-treatment intervals (≤ 1 year, 1–2 years, > 2 years), with only minor variations in effect estimates (Supplementary Table 2).

## Discussion and conclusion

This large, multicentre, real-world cohort study examined whether GTV-CTV1 margin size independently influences radiation-induced dysphagia beyond its impact on OAR doses. Using longitudinal data up to 5 years post-treatment, an ordinal logistic mixed-effects model was developed to predict dysphagia severity over time. GTV-CTV1 margin size was not significantly associated with dysphagia risk, suggesting that margin size in itself does not confer added toxicity beyond its influence on dose to the OAR. However, larger margins were associated with higher mean doses to the middle and lower PCMs, indicating that margin decisions may still indirectly affect toxicity (Table 3).

**Table 3:** Mean doses to organs at risk by margin group.

OAR	< 2.5 mm	2.5–7.5 mm	> 7.5 mm
**PCM upper**	52.9 (ref.)	53.2 (0.99)	48.9 (≤ 0.001)
**PCM middle**	56.3 (ref.)	58.8 (≤ 0.001)	59.1 (≤ 0.001)
**PCM lower**	51.8 (ref.)	51.4 (0.99)	55.9 (≤ 0.001)
**Oral cavity**	41.1 (ref.)	39.6 (0.046)	36.7 (≤ 0.001)

PCM: pharyngeal constrictor muscle; OAR: organ at risk

All values are mean doses in Gy (*p*-value). *P*-values arrived from Wilcoxon rank-sum test.

Importantly, margin size was likely not assigned randomly. In clinical practice, smaller margins may have been deliberately chosen for patients with larger or more advanced tumours due to concerns about treatment tolerance or expected toxicity, reflecting clinician judgement. Such decisions introduce the possibility of confounding by indication, potentially masking a true association between margin size and dysphagia risk. The observed lack of a monotonic relationship between margin size and OAR dose further supports the notion that other planning-related or tumour-specific factors influenced both margin selection and dose distribution.

This complexity is further heightened by variation in the definition and extent of other target volumes, particularly CTV2. While national guidelines recommended that CTV2 encompass an additional 5 mm margin around CTV1, in practice, it often included entire anatomical regions (e.g. the full tongue base if involved), and these patterns may have varied between clinicians and time periods. Thus, such variation may further contribute to the observed lack of a direct correlation between CTV1 margin size and dose to OAR.

To further explore the potential impact of time since therapy, stratified analyses by post-treatment intervals (≤ 1 year, 1–2 years, and > 2 years) were conducted. The overall associations remained consistent across time frames, and the model’s discriminative performance (AUC) was stable (see Supplementary Table 2), supporting the robustness of this study’s findings across different time points.

Several studies from the Netherlands have shown toxicity reductions with smaller margins: Al-Mamgani et al. reported significantly lower acute and late toxicities with smaller combined GTV-CTV-PTV margins, and Navran et al. similarly demonstrated lower dysphagia incidence with smaller CTV-PTV margins, along with reduced OAR doses [8, 24]. Similarly, Zhou et al. found that level IIb CTV optimisation in nasopharyngeal carcinoma patients resulted in significantly lower rates of late xerostomia and dysphagia, with reduced mean doses to salivary glands and PCMs while maintaining survival outcomes [25]. Differences between these studies and this study, such as the use of predefined, standardised margin sizes and consistent use of daily image guidance in previous studies versus more heterogeneous, real-world practices in this cohort, could explain the discrepancies.

While smaller margins would generally be expected to reduce dose to OARs and thereby lower dysphagia risk, this study’s findings suggest that this relationship is not uniform, likely due to the complex interplay between margin size and other tumour- and treatment-related factors, including tumour location, size, anatomical variability, and differences in planning strategies.While the GTV-CTV1 margin was not independently associated with dysphagia risk in this study, it cannot be ruled out that margin size contributes in more complex or indirect ways. Dysphagia is a multifactorial complication, and its occurrence may not be fully explained by the delineated OARs typically included in normal tissue complication probability (NTCP) models [26]. For instance, xerostomia, another common radiation-induced toxicity, often exacerbates swallowing difficulties, making dysphagia even more complex to model accurately. It is plausible that the additional irradiated volume represented by a larger margin could influence structures or mechanisms not explicitly accounted for. Since the predictive model included mean doses to specific OARs, which likely represent more proximate predictors of toxicity, these variables may have attenuated any residual effect of margin size. This again highlights that margin size alone is insufficient to predict toxicity risk without also considering both the distribution of radiation dose and its indirect effects on related toxicities such as xerostomia.

Although chemotherapy was not significantly associated with dysphagia risk (OR 0.71, 95% CI 0.62–1.01, *p* = 0.054), a trend toward a protective effect was observed. This contrasts with previous studies, which have typically identified chemotherapy as a risk factor for dysphagia, in line with the biological understanding of additive toxicity from combined-modality treatment [6]. The borderline-significant observed trend may reflect selection bias rather than a true protective effect. In clinical practice, patients selected for chemotherapy are often younger, have better performance status, and fewer comorbidities – factors that may reduce their overall susceptibility to treatment-related side effects, including dysphagia. Conversely, more frail patients may be spared chemotherapy due to concerns about toxicity, yet still experience higher dysphagia risk due to other underlying vulnerabilities. Thus, the observed protective effect may reflect differences in baseline patient characteristics rather than a true protective role of chemotherapy. Notably, in the statistical analysis, chemotherapy was considered a universal covariate despite the fact that different treatment regimens were used, however, primarily weekly cisplatin. Similarly, nimorazole was associated with a strong and statistically significant protective effect (OR 0.31, 95% CI 0.17–0.56). There is no biological rationale to suggest that nimorazole would reduce dysphagia risk, and as with chemotherapy, this finding likely reflects selection bias. Patients eligible for nimorazole are generally in good clinical condition, as frailer patients are often excluded due to concerns about tolerability. Therefore, the observed association may again be confounded by underlying patient characteristics rather than a true causal relationship.

A consideration in the challenge of accurately capturing treatment-related toxicity. Dysphagia was assessed using physician-reported outcomes, which may introduce observer bias [27]. While structured and clinically grounded, these assessments may not fully capture patients’ subjective experiences of swallowing difficulties. Follow-up timing also varied, raising concerns about potential bias; however, the analysis showed a U-shaped pattern between visit frequency and dysphagia severity, suggesting no clear systematic bias. Notably, this cohort outside of clinical trials likely represents a broader and more clinically diverse population than typical trial settings, thereby strengthening the generalisability of the findings.

The use of real-world data adds important clinical relevance, but also introduces uncertainties. Cohorts from treatments in daily practice are inherently heterogeneous, and despite efforts to include all eligible patients, exclusions were necessary when AI-based segmentation failed or when dysphagia assessments were missing. These exclusions were unlikely to be completely random and may have introduced selection bias; however, the number of excluded patients was small. For example, patients with poor prognosis may have been more likely to discontinue follow-up early, resulting in missing dysphagia data.

Nonetheless, this study has several notable strengths. It utilised real-world, unselected patient data across multiple centres, enhancing the generalisability of the findings. The use of the Danish civil registration system (CPR) and the DAHANCA database ensured high data completeness and follow-up accuracy. The large cohort size enabled robust statistical analyses and meaningful subgroup evaluations. Importantly, the study modelled dysphagia trajectories longitudinally across multiple time points rather than relying on single-point or peak toxicity assessments, offering a more nuanced understanding of dysphagia development over time. Additionally, the predictive model demonstrated strong discriminative performance. Although the AUC alone provides limited clinical value, the observed high discriminative ability combined with well-calibrated predictions underscores the model’s reliability for practical risk estimation. These findings, validated across multiple centres and in an external test split, imply generalisability to other HN cancer populations treated under comparable protocols. Finally, AI-based segmentation of OARs allowed for consistent and standardised contouring across the cohort.Looking ahead, prospective studies, ideally randomised controlled trials, are needed to determine the clinical safety and benefit of GTV-CTV1 margin reduction strategies and to better understand their role in mitigating late radiation-induced dysphagia. The present study contributes to the foundation for an upcoming trial, DAHANCA41: A national randomised non-inferiority study using 0 vs. 5 mm high-dose CTV margin for the primary RT of HNSCC.

In conclusion, the logistic ordinal mixed-effects model identified radiation doses to the oral cavity and lower PCM as important modifiable predictors of radiation-induced dysphagia. The GTV-CTV1 margin provided no additional predictive value beyond these doses and clinical factors, such as tumour volume, although margin reductions may indirectly reduce dysphagia risk by sparing relevant OARs. The model demonstrated good calibration and discrimination for predicting both grade 2+ and grade 3+ dysphagia, supporting its reliability for clinical risk estimation.

## Supplementary Material



## Data Availability

The data used in this study contain sensitive patient information and are not publicly available due to restrictions imposed by the General Data Protection Regulation (GDPR). Access to the data is limited to authorised researchers through institutional agreements and ethical approvals. Requests for data access may be considered on a case-by-case basis and require appropriate approvals from relevant data protection authorities and ethics committees.
