# Supplementary material for: Impact of GTV-CTV margin and other predictors on radiation-induced dysphagia in head and neck cancer patients from DAHANCA group
Source: Acta Oncol. 2025 Sep 18;64:44021. doi: 10.2340/1651-226X.2025.44021 (PMC12452032; doi:10.2340/1651-226X.2025.44021)

Supplementary material has been published as submitted. It has not been copyedited, or typeset by Acta Oncologica

Supplementary Materials

Supplementary Figure 1: Histogram of GTV-CTV1 margins

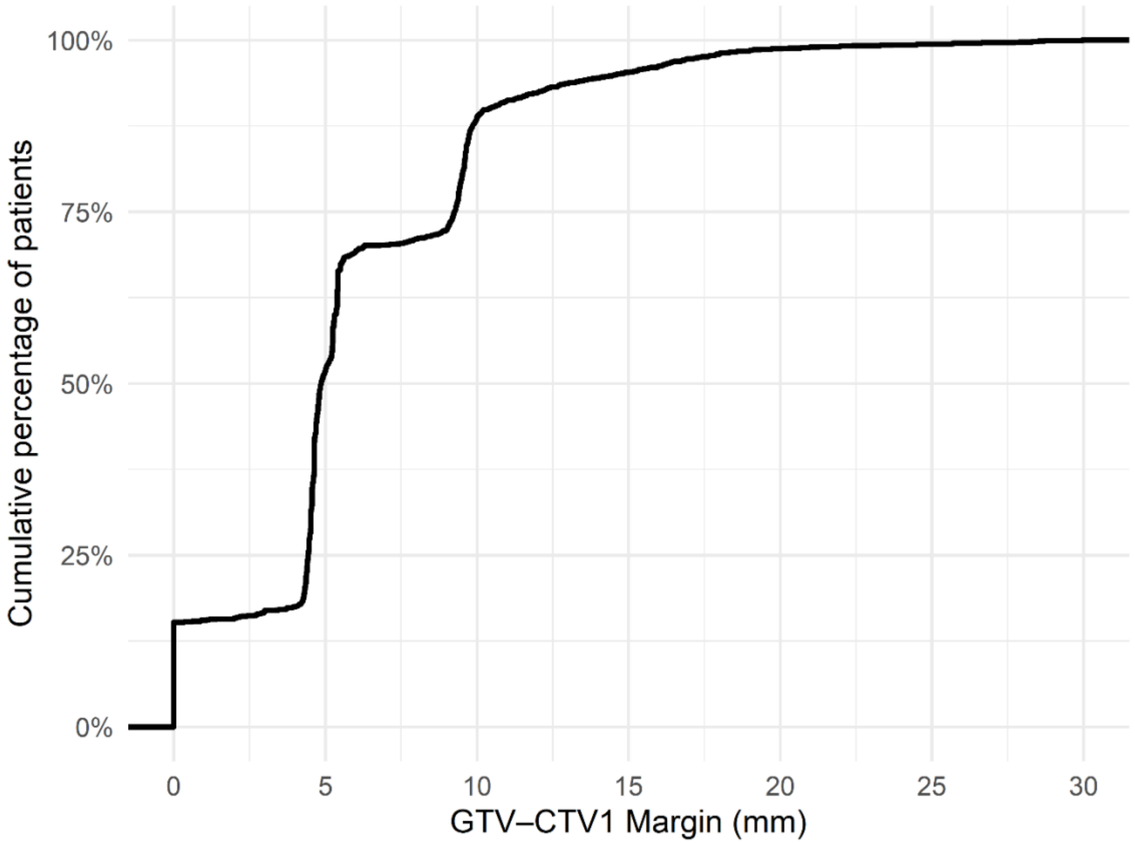

Empirical cumulative distribution function (ECDF) of GTV-CTV1 margin size.
The plot displays the cumulative percentage of patients as a function of GTV-CTV1 margin size in millimetres. Approximately 80% of patients had a margin of 10 mm or less, while margins greater than 20 mm were rare.

Supplementary Figure 2: Calibration plot: Dysphagia grade 2+ and 3+ on train data

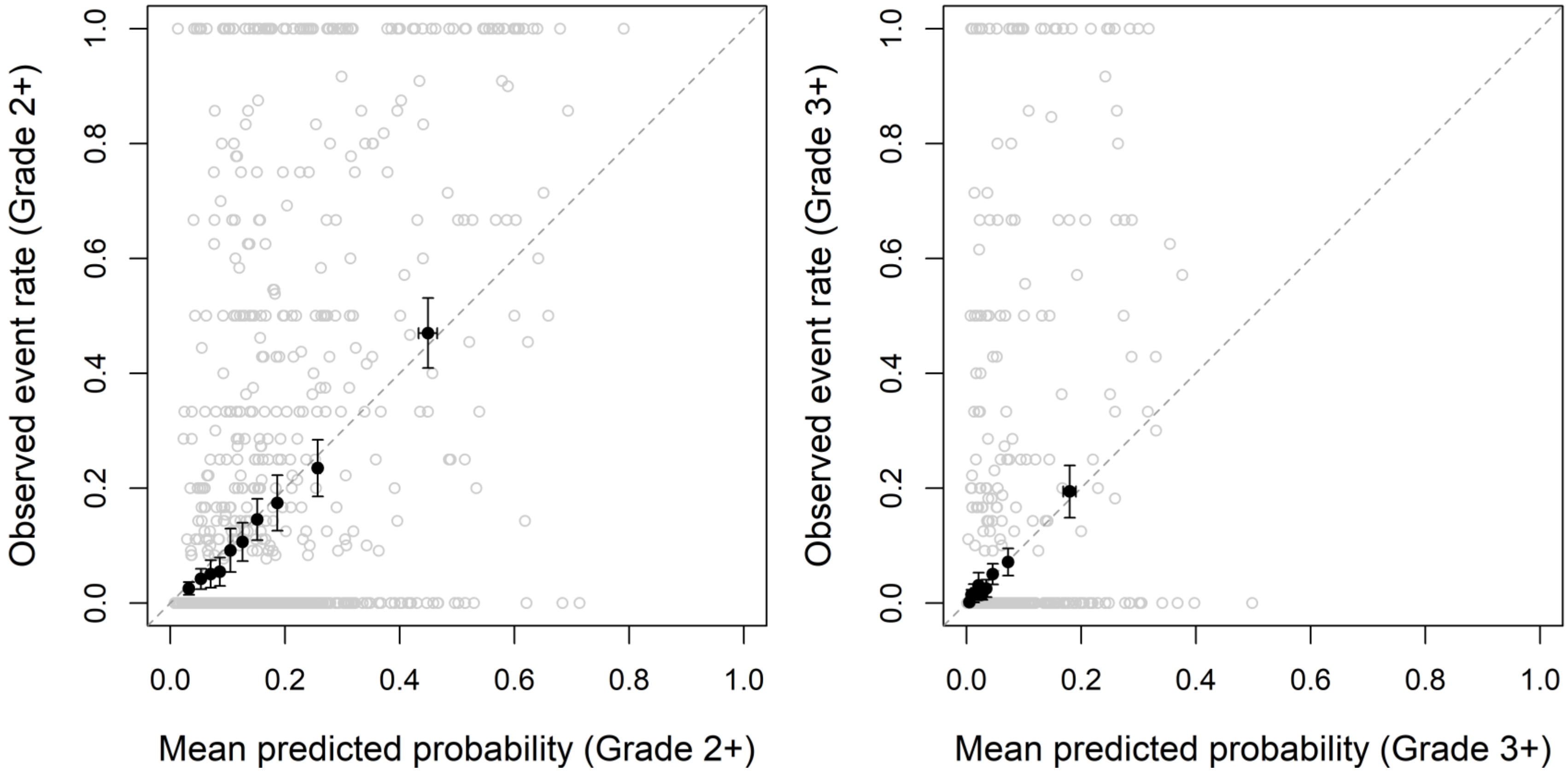

Calibration plot predicting dysphagia grade 2+ and 3+ on training data.
The patients were grouped into 10 equally sized groups (filled black circles) and 95% confidence intervals are displayed in the error bars. Open grey circles represent raw data. Because dysphagia was assessed repeatedly over time, each patient's outcome is expressed as the proportion of follow-up visits with grade ≥2 dysphagia. For example, a patient with dysphagia at 1 out of 4 visits would appear at 0.25 on the y-axis.

Supplementary Table 1: STROBE checklist

	Item No	Recommendation	Page No
**Title and abstract**	1	(*a*) Indicate the study's design with a commonly used term in the title or the abstract	3
		(*b*) Provide in the abstract an informative and balanced summary of what was done and what was found	3
**Introduction**			
Background/rationale	2	Explain the scientific background and rationale for the investigation being reported	4-5
Objectives	3	State specific objectives, including any prespecified hypotheses	5-6
**Methods**			
Study design	4	Present key elements of study design early in the paper	6
Setting	5	Describe the setting, locations, and relevant dates, including periods of recruitment, exposure, follow-up, and data collection	6-7
Participants	6	(*a*) Give the eligibility criteria, and the sources and methods of selection of participants. Describe methods of follow-up	6-7
		(*b*) For matched studies, give matching criteria and number of exposed and unexposed	
Variables	7	Clearly define all outcomes, exposures, predictors, potential confounders, and effect modifiers. Give diagnostic criteria, if applicable	7-8
Data sources/ measurement	8*	For each variable of interest, give sources of data and details of methods of assessment (measurement). Describe comparability of assessment methods if there is more than one group	7-8
Bias	9	Describe any efforts to address potential sources of bias	7-8
Study size	10	Explain how the study size was arrived at	6-7
Quantitative variables	11	Explain how quantitative variables were handled in the analyses. If applicable, describe which groupings were chosen and why	10

Statistical methods	12	(*a*) Describe all statistical methods, including those used to control for confounding	8-10
		(*b*) Describe any methods used to examine subgroups and interactions	10
		(*c*) Explain how missing data were addressed	7-9
		(*d*) If applicable, explain how loss to follow-up was addressed	9
		(*e*) Describe any sensitivity analyses	NA
**Results**			
Participants	13*	(a) Report numbers of individuals at each stage of study—eg numbers potentially eligible, examined for eligibility, confirmed eligible, included in the study, completing follow-up, and analysed	Figure1
		(b) Give reasons for non-participation at each stage	Figure1
		(c) Consider use of a flow diagram	Figure1
Descriptive data	14*	(a) Give characteristics of study participants (eg demographic, clinical, social) and information on exposures and potential confounders	Table1
		(b) Indicate number of participants with missing data for each variable of interest	Table1
		(c) Summarise follow-up time (eg, average and total amount)	11 + Table2
Outcome data	15*	Report numbers of outcome events or summary measures over time	11 + Table3

Main results	16	(*a*) Give unadjusted estimates and, if applicable, confounder-adjusted estimates and their precision (eg, 95% confidence interval). Make clear which confounders were adjusted for and why they were included	12 + Figure2
		(*b*) Report category boundaries when continuous variables were categorized	10
		(*c*) If relevant, consider translating estimates of relative risk into absolute risk for a meaningful time period	NA
Other analyses	17	Report other analyses done—eg analyses of subgroups and interactions, and sensitivity analyses	10
**Discussion**			
Key results	18	Summarise key results with reference to study objectives	12
Limitations	19	Discuss limitations of the study, taking into account sources of potential bias or imprecision. Discuss both direction and magnitude of any potential bias	16
Interpretation	20	Give a cautious overall interpretation of results considering objectives, limitations, multiplicity of analyses, results from similar studies, and other relevant evidence	14-16
Generalisability	21	Discuss the generalisability (external validity) of the study results	16
**Other information**			
Funding	22	Give the source of funding and the role of the funders for the present study and, if applicable, for the original study on which the present article is based	18

*Give information separately for exposed and unexposed groups.

Supplementary Table 2: **Subgroup analysis of predictive performance across post-treatment intervals**

Time window	*AUC (95% CI)*
1 year	
Train 2+	*0.77 (0.74-0.79)*
Train 3+	*0.82 (0.78-0.85)*
Test 2+	*0.75 (0.70-0.79)*
Test 3+	*0.80 (0.75-0.86)*
1-2 years	
Train 2+	*0.77 (0.74-0.81)*
Train 3+	*0.78 (0.72-0.85)*
Test 2+	*0.78 (0.73-0.83)*
Test 3+	*0.82 (0.75-0.89)*
2+ years	
Train 2+	*0.77 (0.73-0.79)*
Train 3+	*0.74 (0.68-0.80)*
Test 2+	*0.77 (0.72-0.82)*
Test 3+	*0.89 (0.83-0.94)*

Supplementary Table 3: **Subgroup analysis by post-treatment intervals: odds ratios**

Variable	*1 year*	*1-2 years*	*2+ years*
GTV to CTV margin (cm)	*0.99 (0.64 – 1.54), 0.979*	*1.00 (0.95 – 1.06), 0.944*	*1.34 (0.83 – 2.16), 0.237*
ln(GTV colume (cm^3))	*1.42 (1.13 – 1.80), 0.003*	*1.34 (0.98 – 1.84), 0.064*	*1.43 (1.10 – 1.86), 0.008*
Smoking status			
Non-smoker	*Ref.*	*Ref.*	*Ref.*
Former smoker	*1.02 (0.59 – 1.78), 0.940*	*1.29 (0.63 – 2.65), 0.489*	*2.08 (1.16 – 3.73), 0.015*
Current smoker	*2.24 (1.26 – 4.00), 0.006*	*3.37 (1.57 – 7.22), 0.002*	*3.80 (2.03 – 7.11), <0.001*

Male sex	*0.49 (0.31 – 0.75), 0.001*	*0.55 (0.31 – 0.98), 0.042*	*0.47 (0.29 – 0.75), 0.002*
Chemotherapy	*0.66 (0.43 – 1.00), 0.053*	*0.81 (0.46 – 1.43), 0.473*	*0.85 (0.52 – 1.39), 0.518*
Nimorazole	*0.35 (0.16 – 0.75), 0.008*	*0.19 (0.06 – 0.63), 0.006*	*0.35 (0.13 – 0.96), 0.042*
Mean dose, oral cavity (per 5 Gy)	*1.48 (1.36 – 1.62), <0.001*	*1.44 (1.28 – 1.62), <0.001*	*1.28 (1.16 – 1.41), <0.001*
Mean dose, lower PCM (per 5 Gy)	*1.34 (1.22 – 1.46), <0.001*	*1.21(1.08 – 1.35), 0.001*	*1.18 (1.07 – 1.30), <0.001*
Baseline dysphagia 2+	*8.92 (4.77 – 16.66), <0.001*	*13.80 (5.75 – 33.13), <0.001*	*10.32 (4.88 – 21.83), <0.001*

All values are presented as odds ratios (95% confidence interval), p-value.

A total of 1,510 patients were included in the 0–1 year post-treatment interval, 1,356 in the 1–2 year interval, and 1,247 in the >2 year interval.

Supplementary Figure 3: Subgroup analysis by post-treatment intervals: Forest plots

Figure 3a: ≤1 year post-treatment

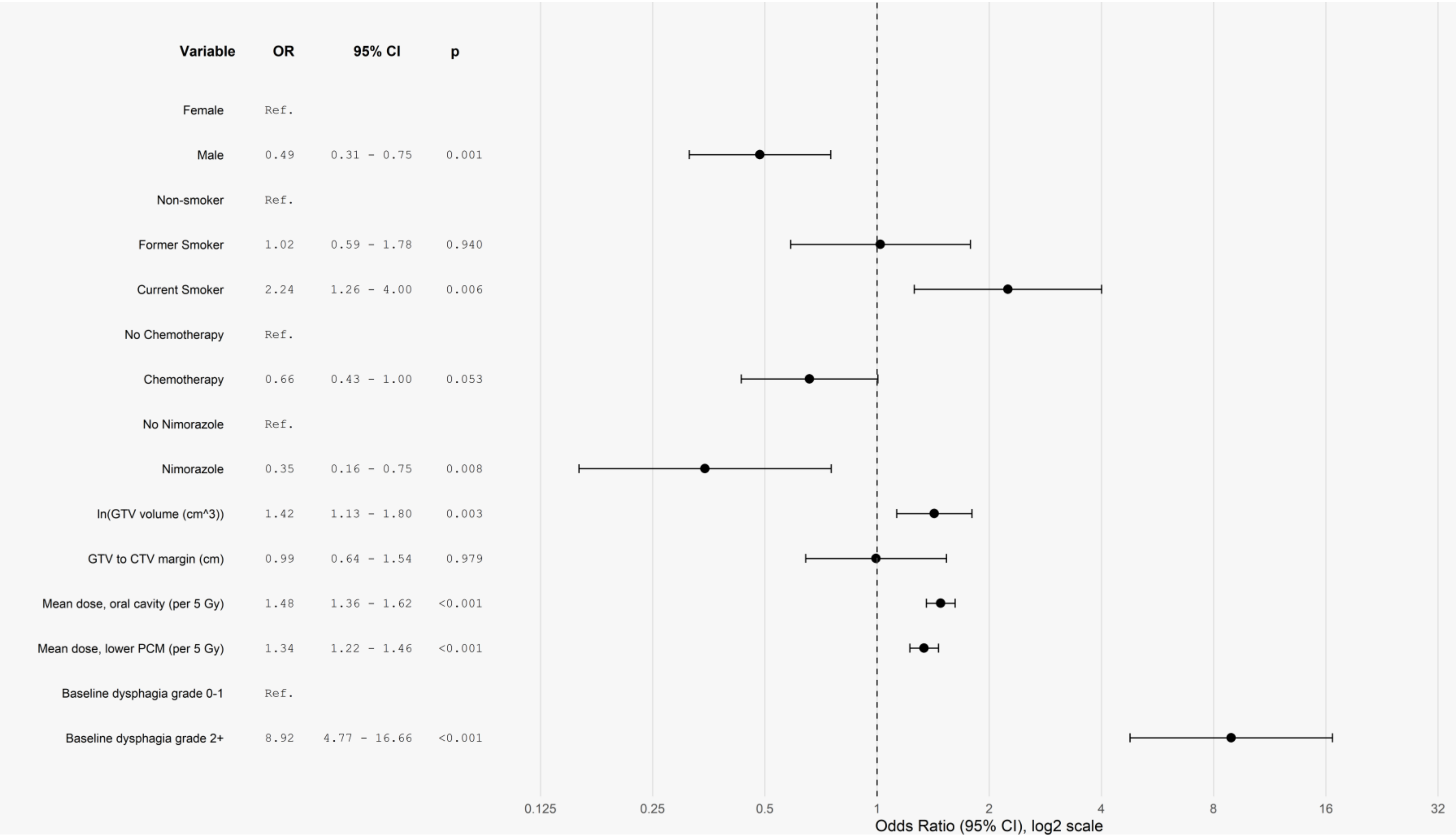

Figure 3b: 1-2 years post-treatment

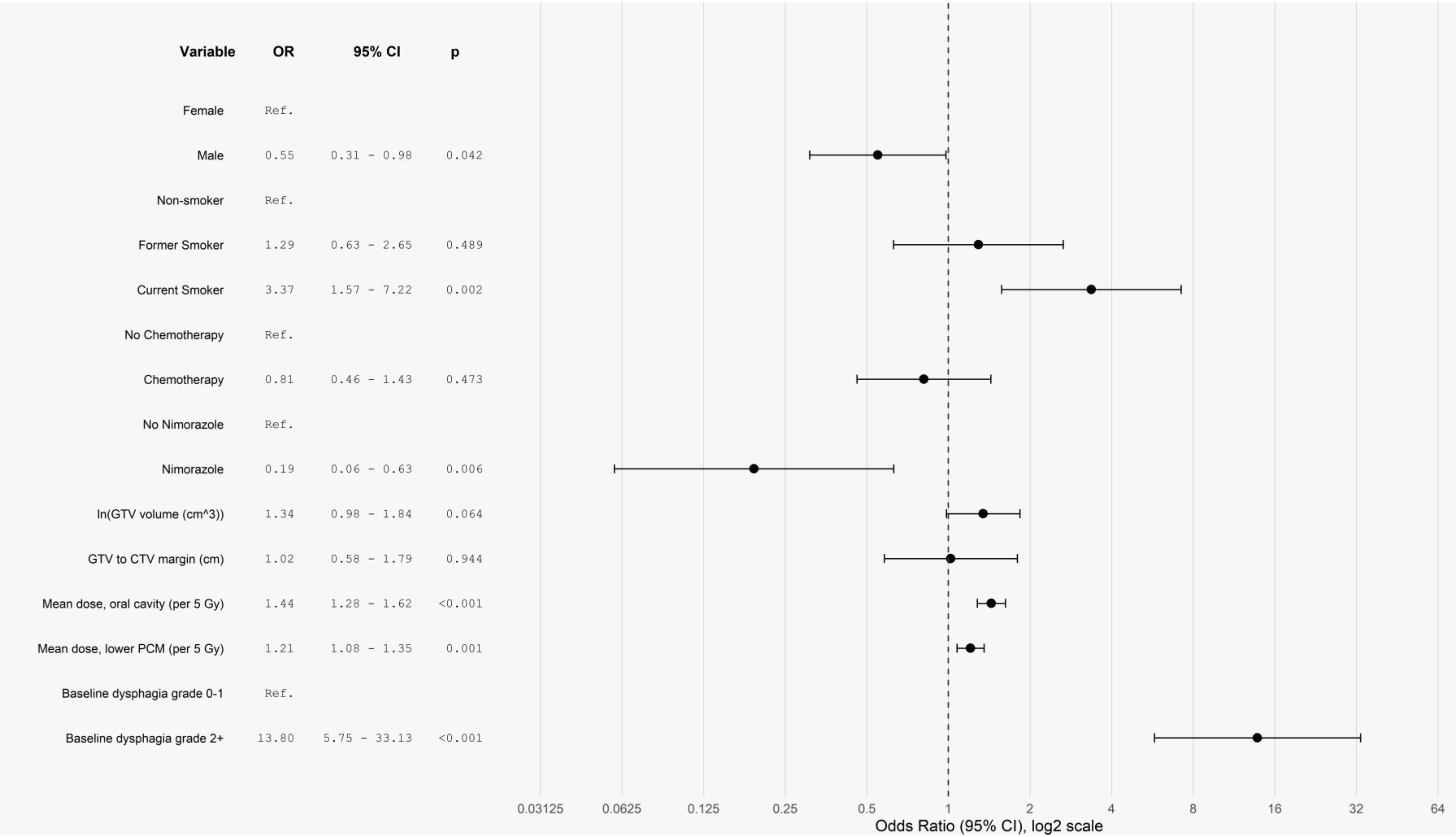

Figure 3c: >2 years post-treatment

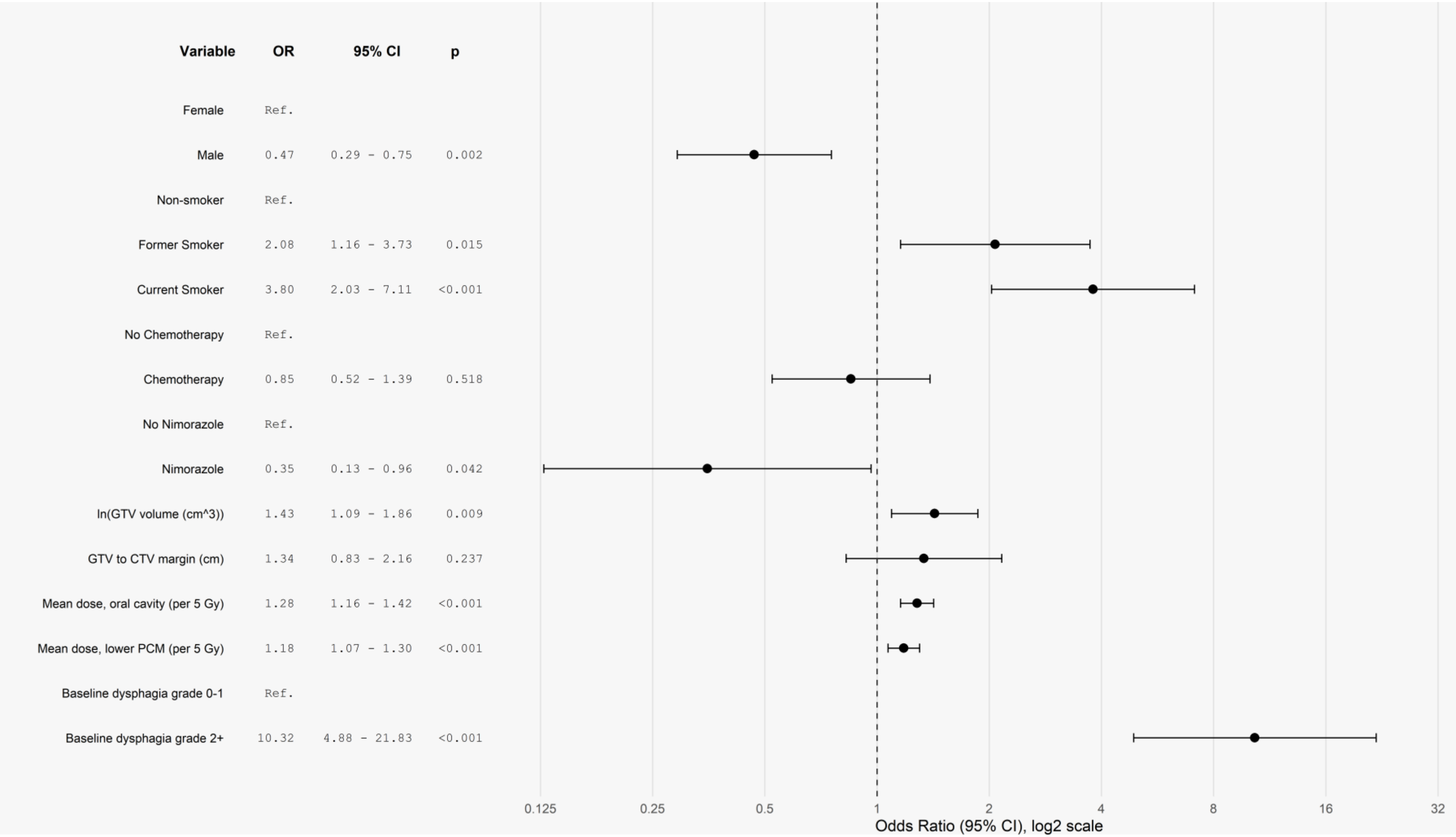

Supplementary Figure 4: **Subgroup analysis by post-treatment intervals: Calibration plots**

Figure 4a: Training set, ≤1 year

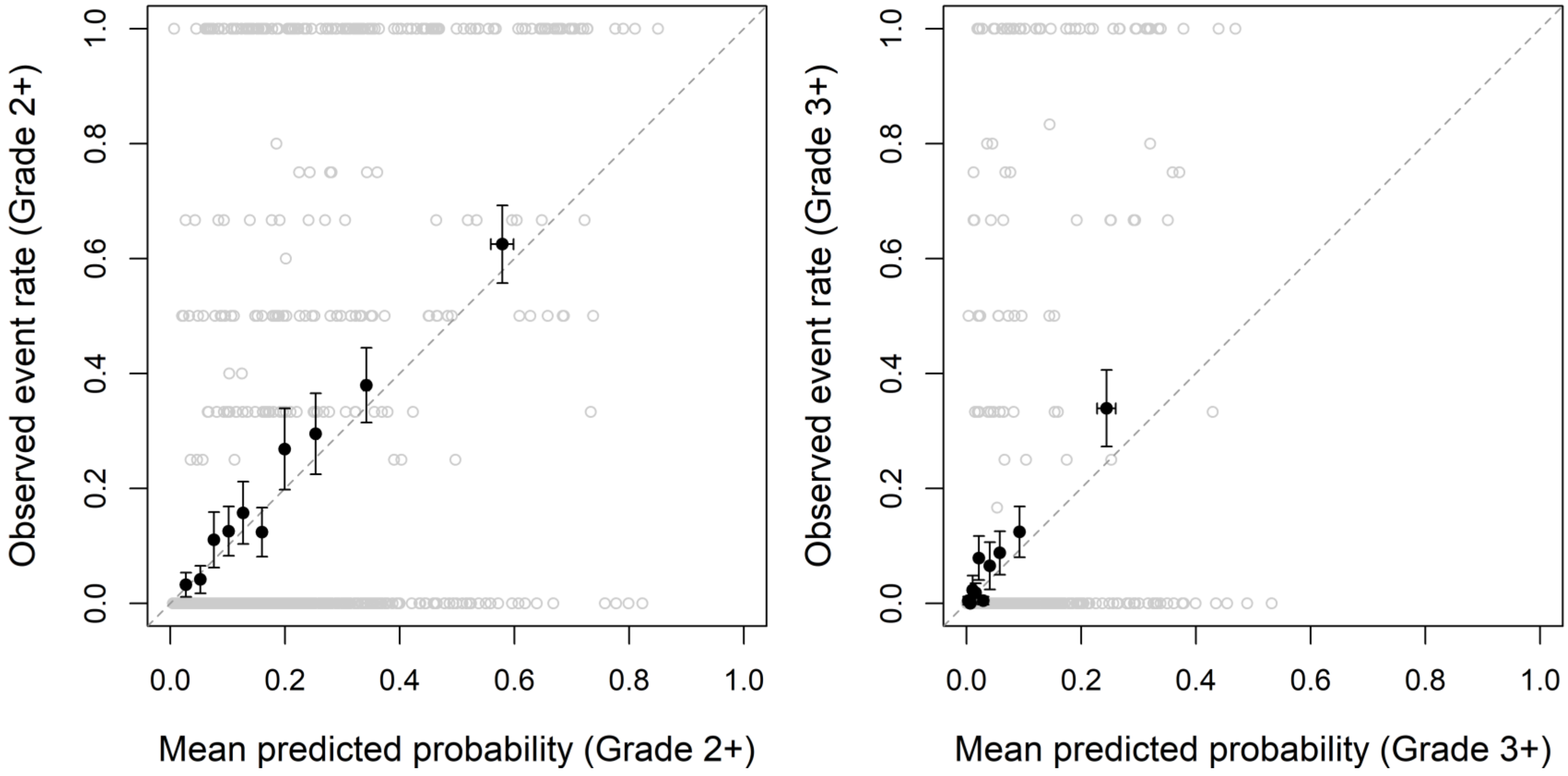

Figure 4b: Test set, ≤1 year

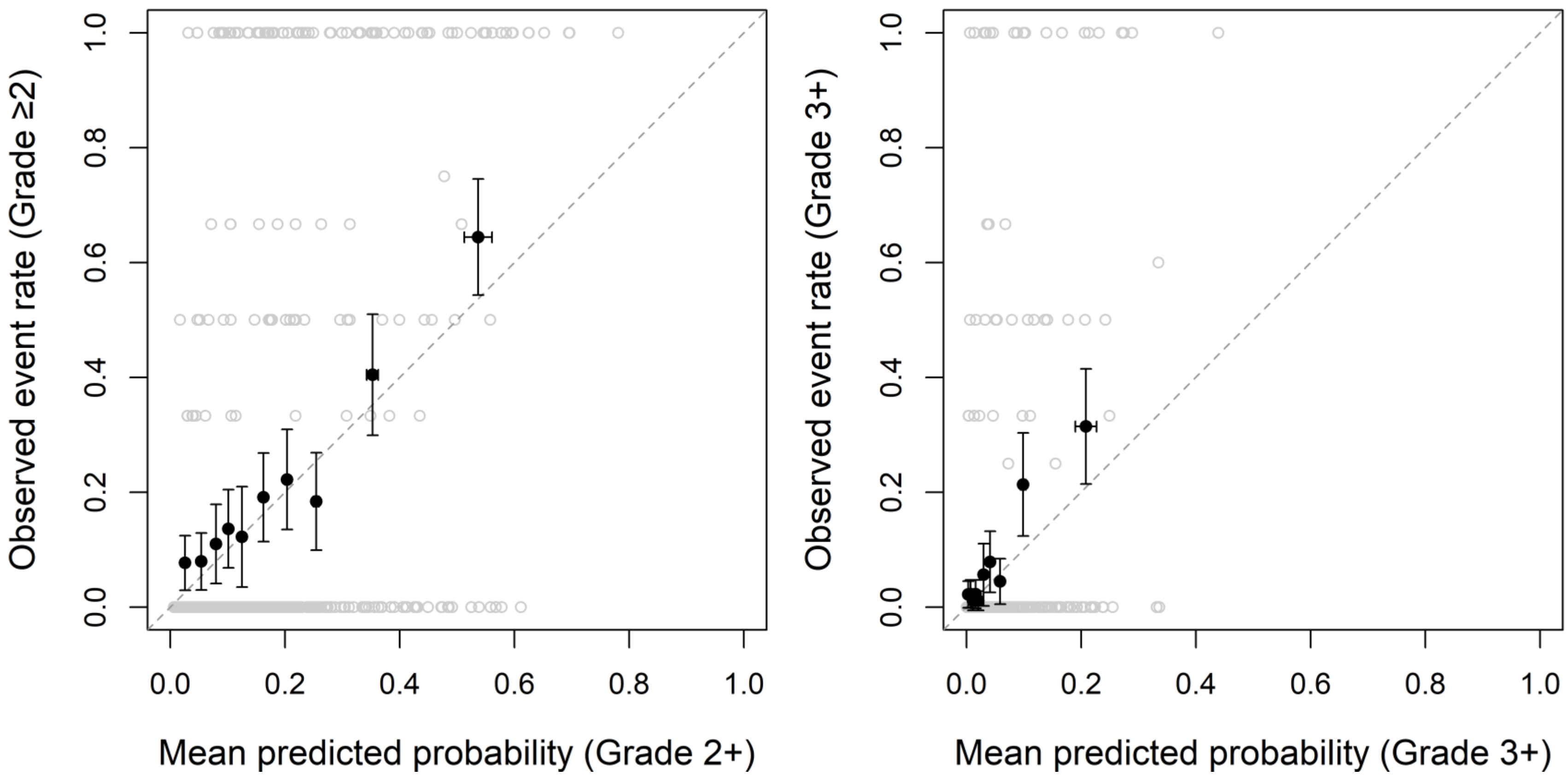

Figure 4c: Training set, 1-2 years

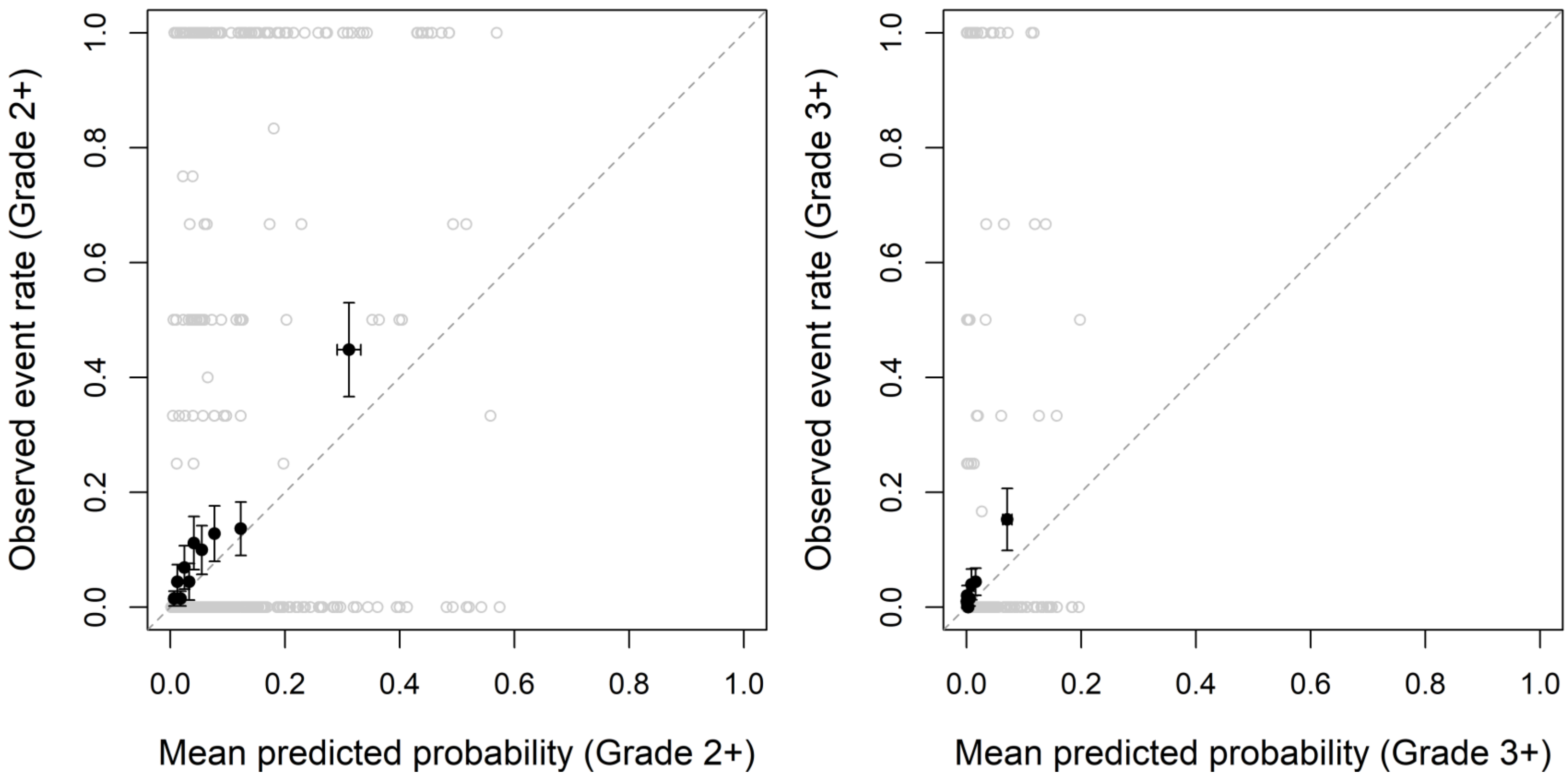

Figure 4d: Test set, 1-2 years

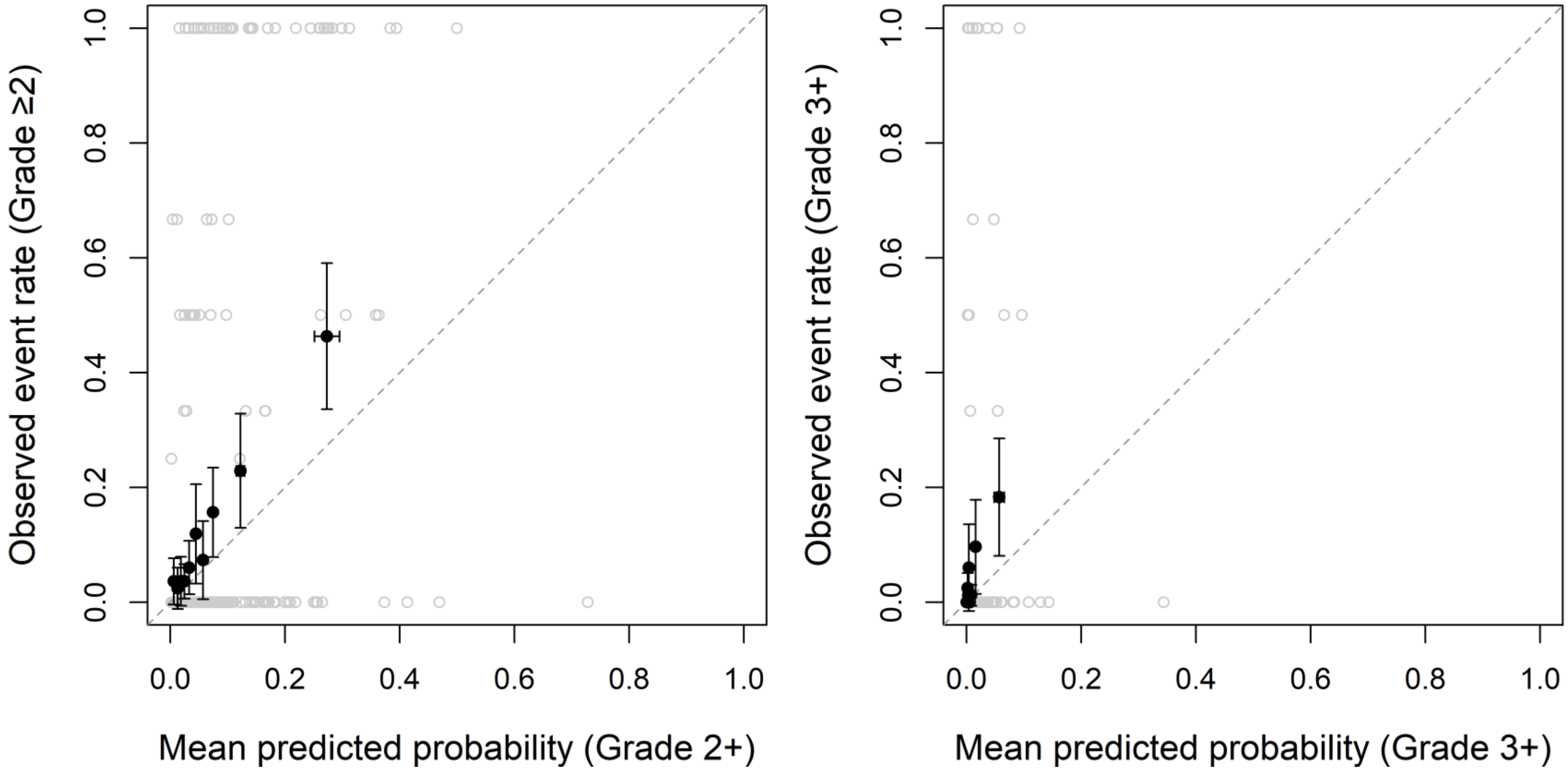

Figure 4e: Training set, >2 years

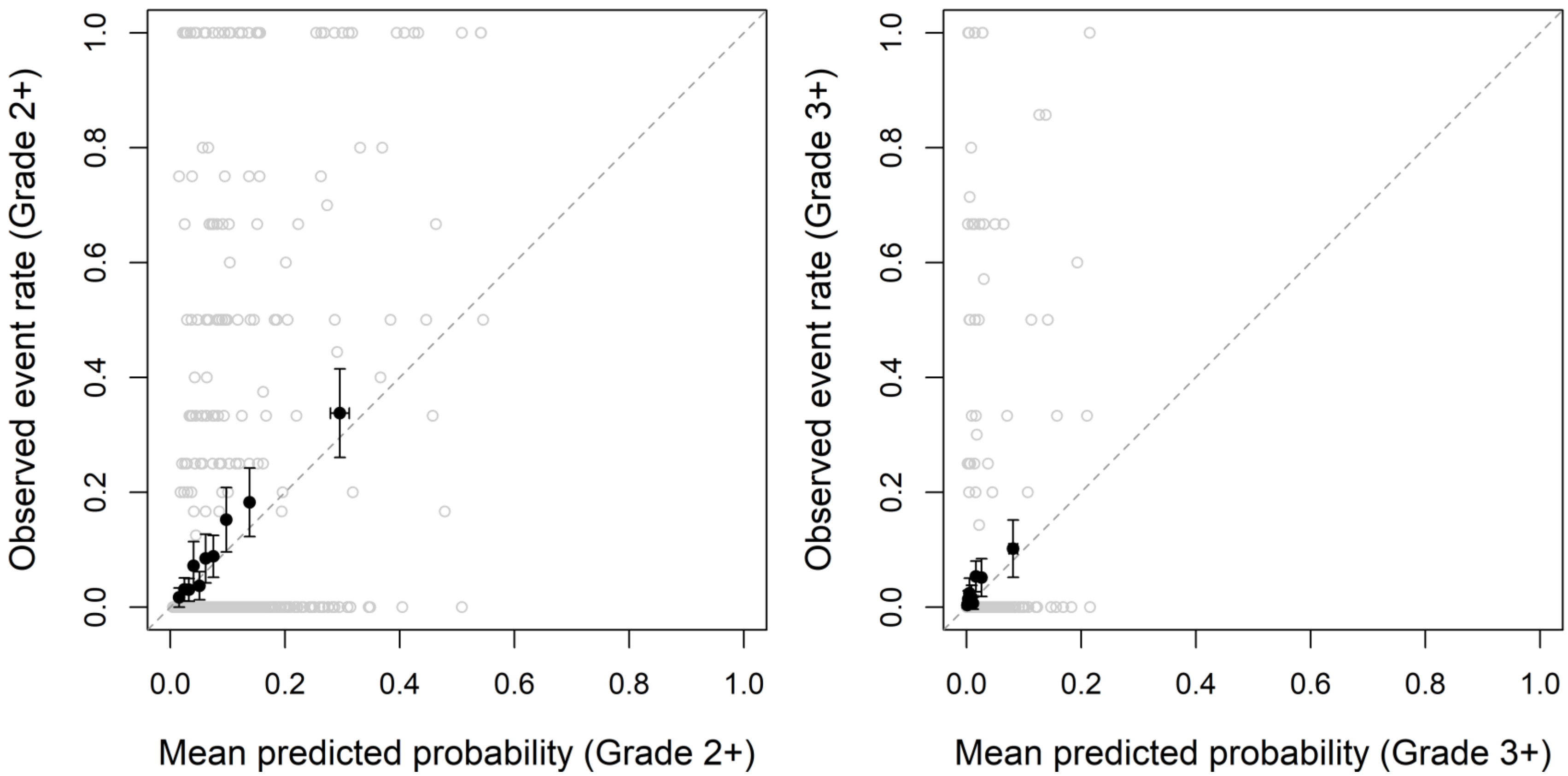

Figure 4f: Test set, >2 years

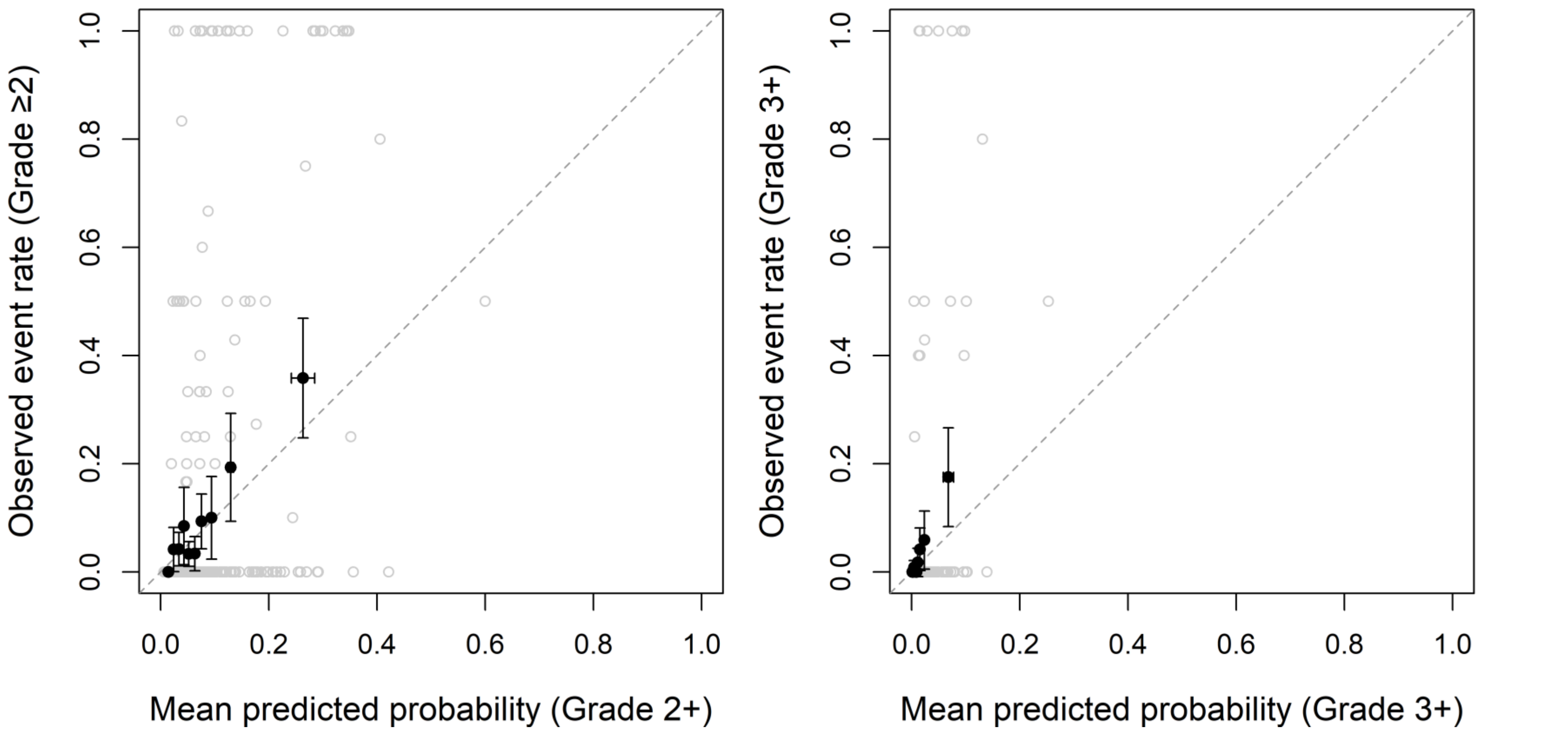

Supplement: Supplementary file 1 [file AO-64-44021-s1.pdf]
